# Assessment of a combination of plasma anti-histone autoantibodies and PLA2/PE ratio as potential biomarkers to clinically predict autism spectrum disorders

**DOI:** 10.1038/s41598-022-17533-0

**Published:** 2022-08-03

**Authors:** Afaf El-Ansary, Mona Al-Onazi, Abdulrahman M. Alhowikan, Mashael A. Alghamdi, Laila Al-Ayadhi

**Affiliations:** 1 Central Research Laboratory, Female Center for Medical Studies and Scientific Section, King Saud University, P.O Box 22452, Riyadh, 11495 Saudi Arabia; 2 Department of Biochemistry, College of Science, King Saud University, Riyadh, Saudi Arabia; 3 Department of Physiology, Faculty of Medicine, King Saud University, Riyadh, Saudi Arabia; 4 Department of Chemistry, Imam Mohammad Ibn Saud Islamic University (IMSIU), P.O. Box. 90950, Riyadh, 11623 Saudi Arabia; 5 Autism Research and Treatment Center, Riyadh, Saudi Arabia

**Keywords:** Biochemistry, Neuroscience, Biomarkers

## Abstract

Autism spectrum disorder (ASD) is a neurodevelopmental disorder characterized by deficiencies in social interaction and repetitive behaviors. Multiple studies have reported abnormal cell membrane composition and autoimmunity as known mechanisms associated with the etiopathogenesis of ASD. In this study, multiple regression and combined receiver operating characteristic (ROC) curve as statistic tools were done to clarify the relationship between phospholipase A2 and phosphatidylethanolamine (PE) ratio (PLA2/PE) as marker of lipid metabolism and membrane fluidity, and antihistone-autoantibodies as marker of autoimmunity in the etiopathology of ASD. Furthermore, the study intended to define the linear combination that maximizes the partial area under an ROC curve for a panel of markers. Forty five children with ASD and forty age- and sex-matched controls were enrolled in the study. Using ELISA, the levels of antihistone-autoantibodies, and PLA2 were measured in the plasma of both groups. PE was measured using HPLC. Statistical analyses using ROC curves and multiple and logistic regression models were performed. A notable rise in the area under the curve was detected using combined ROC curve models. Additionally, higher specificity and sensitivity of the combined markers were documented. The present study indicates that the measurement of the predictive value of selected biomarkers related to autoimmunity and lipid metabolism in children with ASD using a ROC curve analysis should lead to a better understanding of the pathophysiological mechanism of ASD and its link with metabolism. This information may enable the early diagnosis and intervention.

## Introduction

So far the etiological mechanisms of Autism Spectrum Disorders (ASD) have not yet been understood and diagnostic and specific early predictive biomarkers for the core features of ASD do not exist. Nevertheless, the effect of early intervention is well recognized and ascertained for this disorder. Up to the increasing prevalence of ASD, the study of diagnostic markers has gained considerable care. ASD is a multifactorial disease that comprises an interaction between genetic and environmental contributors^1^.

Membrane lipids are exceptionally important to life, mainly because they offer four important functions for cellular health^2^. They offer:(1) the matrix for all cellular membranes, allowing compartementation of enzymatic and chemical reactions within the cell (2) energy storage pools; (3) providing bioactive molecules that are used in diverse signal molecular recognition and transduction pathways; and (4) functional molecules that interact with other membrane components, such as proteins and glycoproteins^3,4^. The formations of lipid domains in the matrix of cellular membranes are principally due to the interactions of glycerolphospholipids, particularly phosphatidylcholine (PC), and phosphatidylethanolamine (PE) together with sphingomyelins^5–7^. Under normal physiological conditions, membrane phospholipids are present in various phases as fluid, semi-solid and solid that is organized into domains characterized by different lipid configurationally arrangements.

Like phosphatidylcholine (PC), the phospholipid phosphatidylethanolamine (PE) is also zwitter ionic, but holds a much smaller head group, both in regards of actual size and hydration, and accordingly, it is thought to weaken head group packing. Up to this, PE was predominantly proposed to enable protein binding to membrane in general^8^. However, in addition to these small changes in the overall packing of membranes, PE also modulates the charge of phosphomonoester-containing membrane lipids.

Phospholipase A2s are enzymes that catalyse the hydrolysis of fatty acid at the sn-2 position of the glycerol backbone of membrane phospholipids (PC, PS, PI, and PE). Given the asymmetric distribution of fatty acids in phospholipids, wherever saturated fatty acids usually present at the sn-1 position, and polyunsaturated fatty acids (PUFA) such as those of the omega-3 and omega-6 series tremendously localize in the sn-2 position, the phospholipase A2 catalytic reaction is of greatest importance as a controlling checkpoint for the mobilization of these fatty acids and the subsequent synthesis of omega-6-derived proinflammatory eicosanoids on one hand under a variety of pathophysiological conditions, and omega-3-derived derived pro-resolving mediators on the other under physiological conditions. Up to this, PLA2s could be considered as a key to membrane phospholipid remodelling reactions, and the generation of distinct lipid mediators with essential functions in biological processes. Understanding the cellular roles of these enzymes depending upon activation conditions could be of great help to clarify the etiological mechanisms of many neurological disorders. In ASD it is well documented that patients have significantly lower PE and higher PLA2, which makes the PLA2/PE ratio a perfect biomarker of pro-inflammation and membrane phospholipid alteration^9,10^.

A large number of serum antibodies directed against useful structures of the cell such as nucleic acid, nuclear molecules, receptors, or other useful cell components can be identified in human autoimmune diseases. Its presence plays a crucial role in the prediction and diagnosis of these diseases. It is well known that patients may carry autoantibodies many years before they manifest clinical presentation of the disease and detection of these antibodies in serum has been shown to have strong predictive value^11^.

Since the discovery of anti-DNA antibodies in systemic lupus erythematosus (SLE) sera, over 50 years ago^12^, specific autoantibodies have been widely studied and defined and a large number of auto-epitopes have been recorded. Histones as an important protein act as wrapper around the DNAand thus play an important role in gene expression regulation^13,14^. The post-translational modifications of histone proteins alter their interaction with DNA and other nuclear proteins and provoke the immunity against self^15^. Although the functional activity of antihistone- autoantibody is largely unknown, they may possess proteolytic activity towards histone proteins^16^. Whether this directly contributes to disease pathogenesis is unclear at this stage^15,16^. Mecocci et al.^17^, reported that in dementias, the presence of anti-histone antibodies might reveal a change of membrane fluidity and integrity with outflow of nuclear immunogens or impairments of immune response. Recently, Nandi et al.^18^, reported that loss of membrane rigidity or increase in the membrane fluidity might remain as one of the serious dynamics linked with the change in membrane properties in many neurological disorders.

Interestingly, membrane fluidity was found to be greatly affected by their interaction with neurotransmitters^19,20^. One of the well-studied neurotransmitters is Gamma-amino-butyric Acid (GABA) which is the main inhibitory neurotransmitter in the brain^21–24^. Up to this, lack of appropriate functioning of GABA leads to numerous neurological disorders among which are ASD^25,26^. Dysfunction of GABAergic signalling was recently related to the increase in the membrane fluidity as etiological mechanism in ASD^18^.

Under physiological conditions, reactive nitrogen and oxygen species are constantly produced. Nevertheless, excess of these radicals may damage biomolecules such as lipids, proteins and nucleic acids^27–29^. These radicals have been strongly associated with ASD phenotypes. Peroxynitrite, an oxidant and nitrating molecule, formed in in vivo, when nitric oxide reacts with superoxide radical. The elevated levels of nitro-tyrosine noticed in tissues affected by autoimmune conditions have been attributed to peroxynitrite-mediated boosted nitration of tyrosine amino acid residue in proteins. Although histone proteins are conserved and weak immunogens, but they display strong immunogenicity after nitration. Rabbits challenged with peroxynitrite-modified histone prompt high titre antibodies, demonstrating that peroxynitrite modification created immunogenic epitopes. This might help to suggest that peroxynitrite-modified histones autoantibodies are contributed in the initiation and progression of autoimmune diseases among which is ASD^15^. Nadeem et al.^30^ proved that BTBR mice as rodent model of ASD have increased lipid/protein oxidation products in cerebellum and neutrophils/CD3 + T cells, raised NADPH oxidase (NOX2) activity, and inducible nitric oxide synthase (iNOS) expression concomitant with decreased levels of antioxidant enzymes and glutathione.

Histones have been shown to bind strongly to anionic phospholipids and are rapidly released during cell injury^31^. Hirai et al.^32^ have demonstrated that phospholipids and cardiolipin can inhibit histone function. Pereira^33^ has postulated an important role of anti-histone antibody in anti-cardiolipin reactions. They suggest the possibility that autoantibodies to histones or to histone-phospholipid complexes may contribute to anti-phospholipid activity. In case of disrupted BBB in autistic patients, the influx of anti-histone antibodies from blood to the brain and its binding to membrane phospholipids could lead to neuronal damage either by direct interaction of these complexes with neurons or by functional impairment due to their interaction with astrocytes, activation of endothelial cells and adherence to different brain cells^34^.

The ROC curve is the most commonly used graphical tool for evaluating the diagnostic value of a biomarker. In ROC curves, the area under the curve (AUC) is an effective way to summarize the overall diagnostic accuracy of the test. AUCs are effective and combined measure of sensitivity and specificity that measures the characteristic validity of a biomarker. It takes values from 0 to 1, where a value of 0 shows an inaccurate test and a value of 1 reflects a perfectly accurate test. In general, an AUC of 0.5 proposes no diagnostic or discrimination value of a biomarker, 0.7 to 0.8 is considered acceptable, 0.8 to 0.9 is considered excellent, and more than 0.9 is considered outstanding ability to diagnose patients with and without the disease or condition based on the ROC analysed biomarker^35^.

Due to the complexity of the brain and pathogenesis of ASD, a combination of several biomarkers could epitomize a more potent approach to diagnose this disorder^36,37^.

The ROC curve is the most common graphical tool for assessing the diagnostic power of a biomarker. For example, our most recent study showed that a combination of nine biomarkers and 5 ratios distinguished autistic children from healthy controls with a high AUC (area under the ROC curve) value^37^.

In this study, it was very interesting to test the possibility to find ROC combination models between twenty four previously published biomarkers presenting different signalling pathways in the plasma of the same group of patients with ASD. Logistic regression was performed between the twenty four variables with all possible permutations, and two models of combined ROC that have significant increase in the AUC of one or more variable were considered Supplementary Table S1. First model was published as combined Alpha-Synuclein, cyclooxygenase-2 and prostaglandins-EP2 receptors as neuroinflammatory biomarkers of ASD^38^. The second model is that presented in the current study showing combination between anti-histone autoantibodies with PLA2/PE relative ratio as a perfect biomarker of pro-inflammation and membrane phospholipid alteration in ASD. Understanding the mechanism behind the combination of anti-histone autoantibodies and PLA2/PE might help to highlight their roles in the etiology of this disorder.

## Methods

### Participants

The study protocol was approved by the ethics committee of medical Collage, King Saud University according to the most recent Declaration of Helsinki (Edinburgh, 2000). Two groups of study participants were enrolled in the study comprising of 40 patients with ASD and 40 age and gender matched healthy control. The inclusion period ranged from May 2019–December 2019. Informed consents were obtained from all participants and signed by their parents. Both groups were recruited through the ART Center (Autism Research & Treatment Center) clinic. The diagnosis of ASD was confirmed in all study subjects using the Autism Diagnostic Interview-Revised(ADI-R) and the Autism Diagnostic Observation Schedule (ADOS) and 3DI (Developmental, dimensional diagnostic interview) protocols. The ages of autistic children involved in the study were 5.5 ± 2.2 years old. All were simplex cases (i.e. family has one affected individual). All are negative for fragile x syndrome gene. They have a well characterised autistic phenotypes with mild-severe recorded values between 30–45.5 Childhood Autism Rating Scale (CARS), moderate-severe Social Responsiveness Scale (SRS) (70–˃ 76), and a well characterized Short Sensory Profile. Forty age- and sex-matched control participants, aged (5.3 ± 2.3) years old were recruited from the Pediatric Clinic at the same hospital. Participants were excluded from the study if they had fragile X syndrome, serious neurological disorders, or any other medical conditions. All participants were screened by way of parental interview for existing or historical physical illness. All patients and controls included in the study were on similar but not identical diet and none of them were on any special gluten restricted diet.

### Blood sampling

After overnight fasting, blood samples were collected from children with ASD and healthy controls by a skilled technician into 3-ml blood collection tubes containing EDTA. Immediately after collection, blood was centrifuged at 4 °C at 3000*g* for 20 min. The plasma was separated, distributed into three 0.5 ml aliquots (to avoid several freeze-thaws cycles) and stored at − 80 °C until use^39^.

### Ethics approval and consent

This work was approved by the ethics committee of King Khalid Hospital, King Saud University (Approval number: 11/2890/IRB). A written consent was obtained from the parents of all participants recruited in the study as per the guidelines of the ethics committee.

### Biochemical assays

Phospholipids was performed on a Kaneur Maxi Star HPLC system with four solvent lines, a degasser SEDEX 55 evaporating light detector (SEDEX 55 Lichtstreu detector, S.E.D.E.E., France) which was coupled with Apex M625 software (Autochrom, USA). As the nebulizing gaz, N2 was used at a flow rate of 4 l/ min, and a nebulizing temperature of 40 °C. The gain was set at 8 and 2.0 bar N2. A 125 × 4.0 mm Si-60 column with 5 μm particle diameter (Lichrosher) was used. The elution program was a linear gradient with 80:19.5:0.5 (V/V) chloroform: methanol: water: ammonia (NH3) at 22 min and the column was allowed to equilibrate until the next injection at 27 min. The injection volume was 50 μl. A liquid phase extraction procedure adapted from the method described by Bligh and Dyer^40^ was used to extract the serum samples. Briefly, 50 μl of each sample was diluted with 750 μl deionized water and mixed well. Then 2 ml of methanol and 1 ml of chloroform were added to the sample and mixed well. Then the mixture was homogenized (Rotary mixture 34,526, Snijders) for 15 min. The mixture was centrifuged for 5 min at 4000 rpm, and 0.5 ml of supernatant was directly used in a 2 ml vial for HPLC analysis^39^. The HPLC instrument was programmed to accommodate 100 vials in one run.

Assay of cPLA2 cPLA2 concentration was measured according to the manufacture’s instruction using a competitive enzyme immunoassay technique a product of Amsbio, Blue Gene Company. The detection range of the product was 1.56–100 ng/ml.

### Assay of antihistone-antibodies

Antihistone- autoantibody was measured using ELISA kit, a product of Genway Biotech, San Diego, USA. The samples were run together, in parallel on the same run with the standards, and controls. After 30 min incubation at 25 °C, antibodies to highly purified human antihistone—specific antibodies, if present in the serum, would bind in the wells. Washing the wells to eliminate the unbound serum antibodies. Horseradish peroxidase enzyme conjugated anti-human IgG immunologically binds to the bound patient antibodies, forming a conjugate/antibody/ antigen complex. When substrate is washed in the presence of bound conjugate, it is hydrolyzed to form a blue colour. The addition of an acid stops the reaction, resulting in a yellow end product. The intensity of this yellow colour was measured photometrically at 450 nm. The amount of colour is directly proportional to the concentration of IgG antibodies present in the control and patients samples. To increase accuracy, all samples were analysed twice in two independent experiments to evaluate inter assay differences and to confirm reproducibility of the detected results^39^.

### Statistical analyses

In this study, IBM SPSS software, version 22.0 (IBM Inc., Armonk, USA) was used to analyze the data. Normality of data for each group was done using Shapiro–Wilk Test, the results are presented as the Minimum, Maximum and Median, and Mann–Whitney Test for comparing two nonparametric groups was used for statistical evaluations, with P ≤ 0.05 considered a significant difference. Correlation between various nonparametric variables was done using Spearman rank correlation coefficient (R). For the combined ROC curves, odds ratios (ORs) obtained from logistic regression analyses describe associations of biomarkers with the clinical status. ROC curves were constructed for each logistic regression model. The area under the curve (ROC-AUC) was compared between each marker and marker combination using a nonparametric method. Similarly, the predictiveness curves, as a complement to ROC curves of the measured parameters, were drawn using a Biostat 16 computer program and the x-axis represents percentile rank of the biomarker, the y-axis represents the probability of identifying the disease, and the horizontal line is the prevalence of the disease. The predictiveness curve represents a spontaneous and graphical tool to compare the predictive power of any measured biomarker.

### Ethics approval and consent to participate

All procedures performed were in accordance with the ethical standards of the institutional and/or national research committee and with the 1964 Helsinki declaration and its later amendments or comparable ethical standards. This work was approved by the ethics committee of King Khalid Hospital, King Saud University (Approval number: 11/2890/IRB).

### Consent for publication

All authors have read the manuscript and agreed for the submission.

## Results

**Figure 1:**
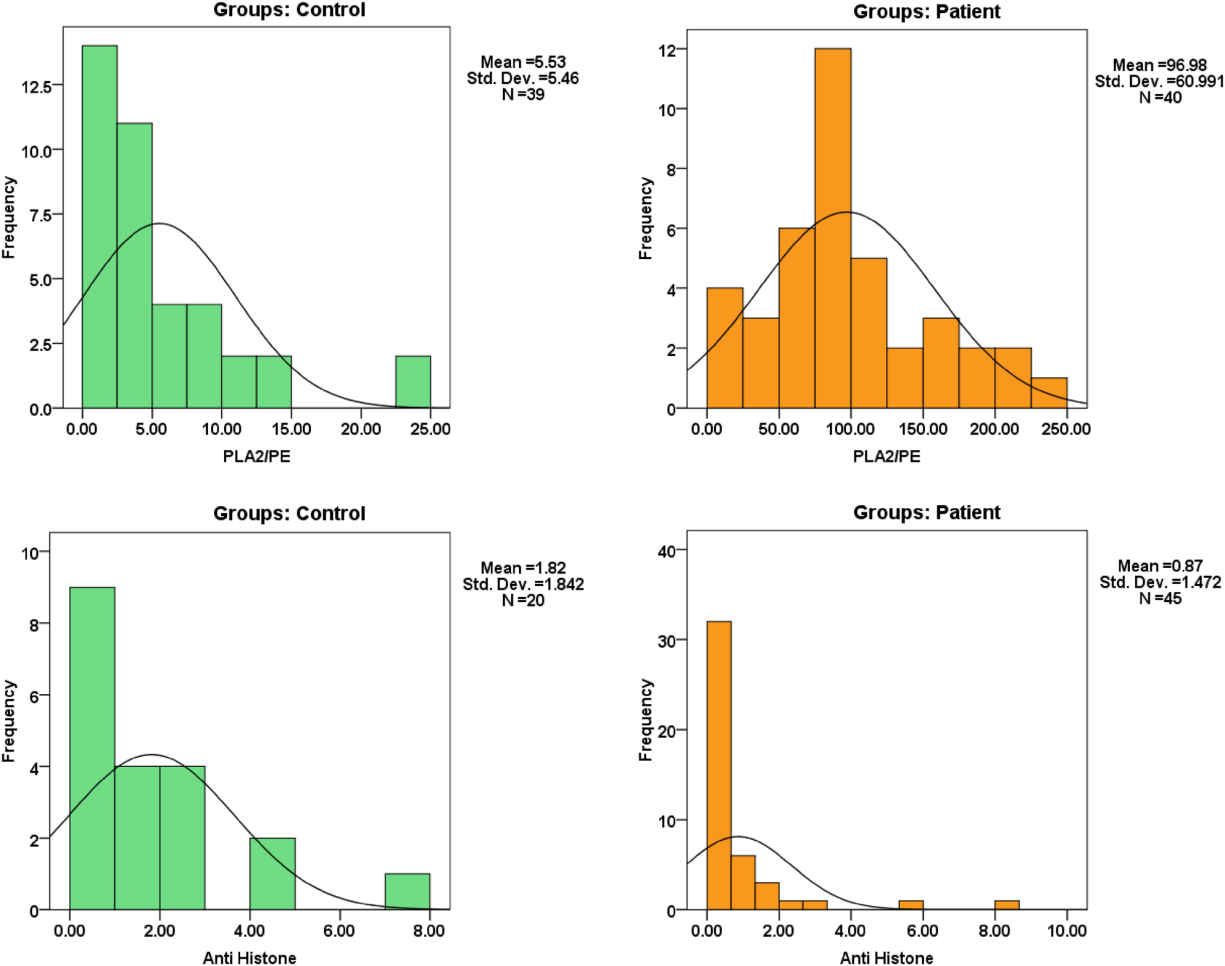
Data distribution of anti-histone autoantibodies and PLA2/PE relative ratio in control and ASD patients.

**Figure 2:**
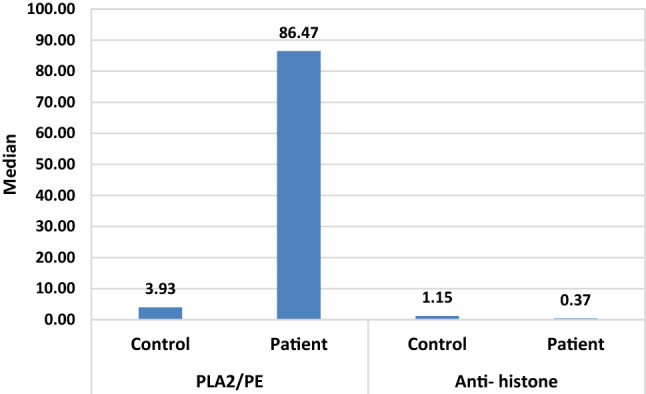
Median of PLA2/PE and anti histone for each goup.

**Figure 3:**
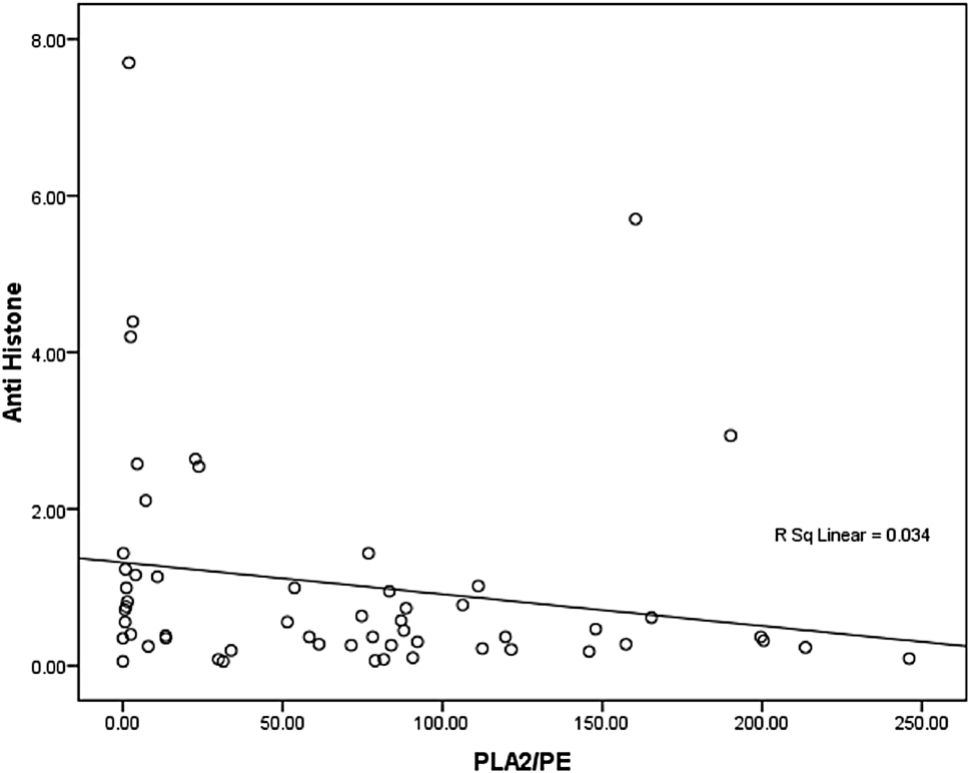
Correlation between PLA2/PE and anti histone with best fit line curve (negative correlation).

**Figure 4:**
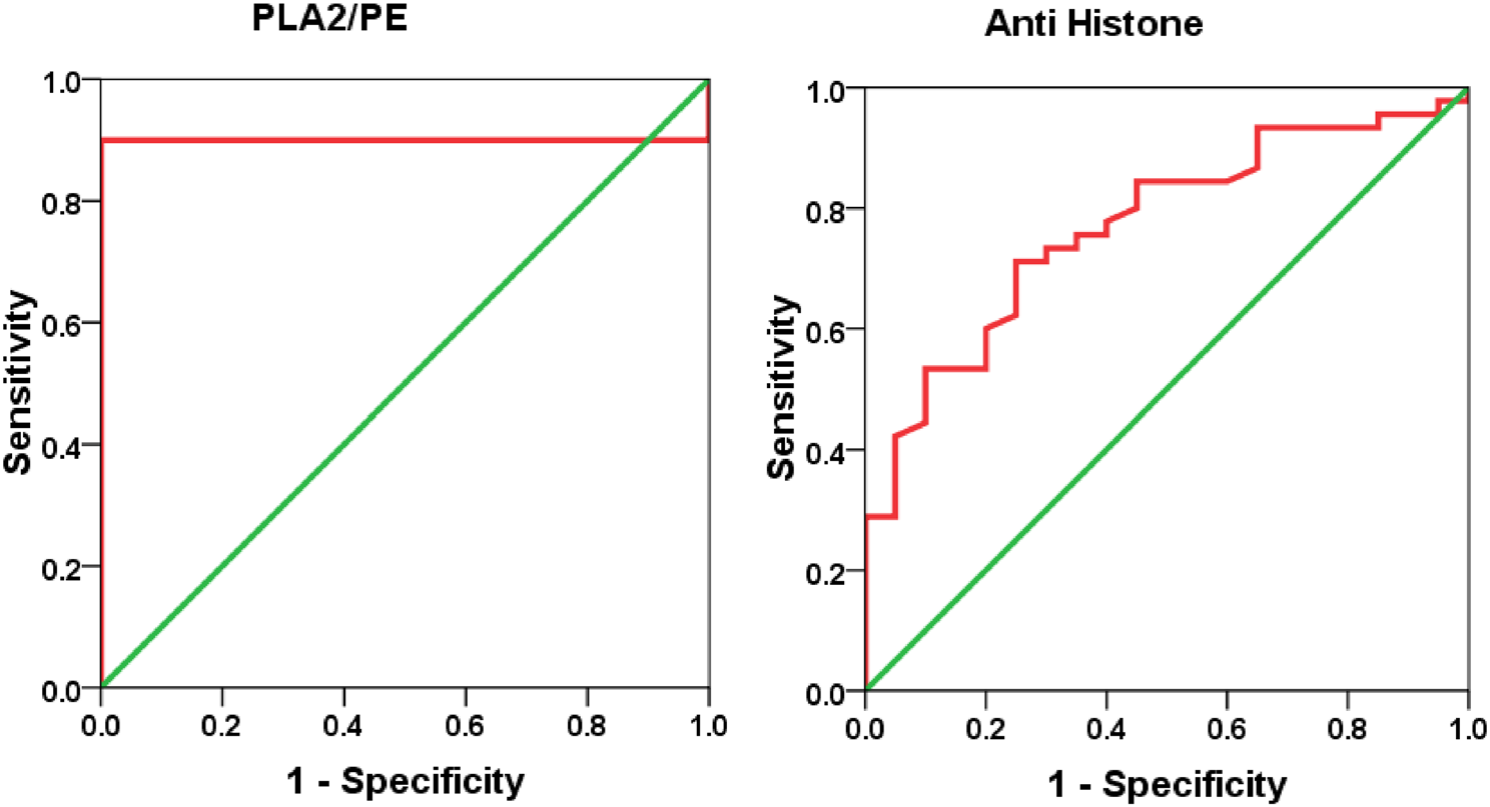
ROC curve of PLA2/PE and anti histone in patient group.

**Figure 5:**
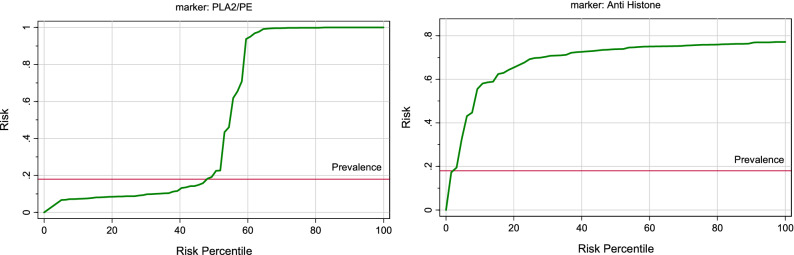
Predictiveness curve of each parameter for patient group.

**Figure 6:**
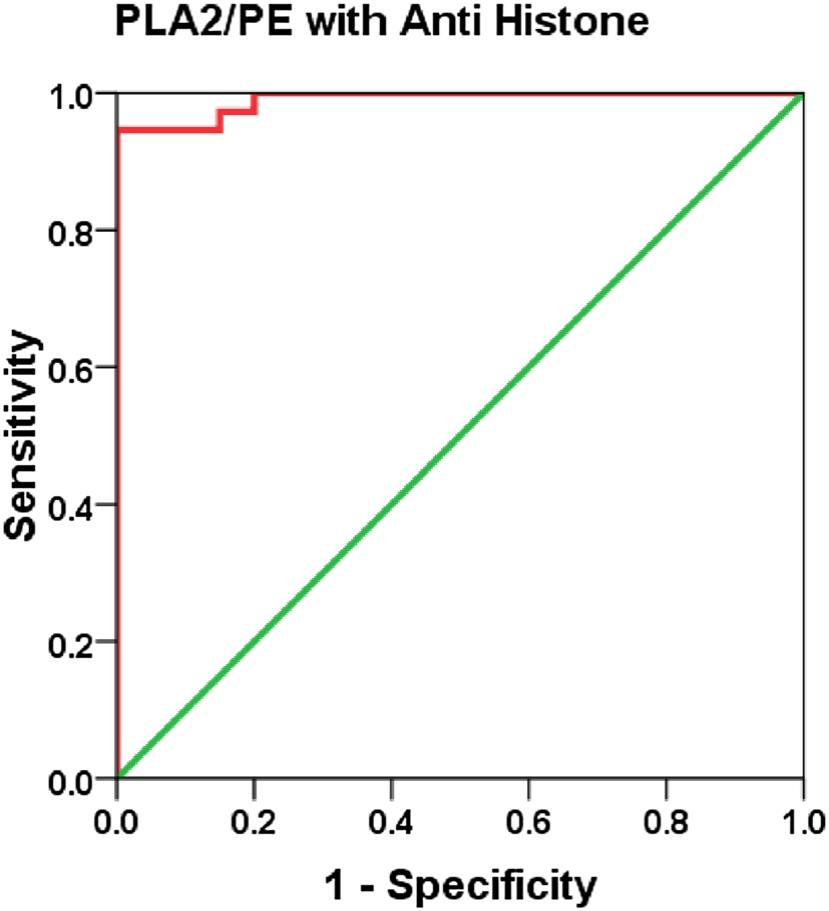
ROC curve of combining two parameters in patient group.

**Figure 7:**
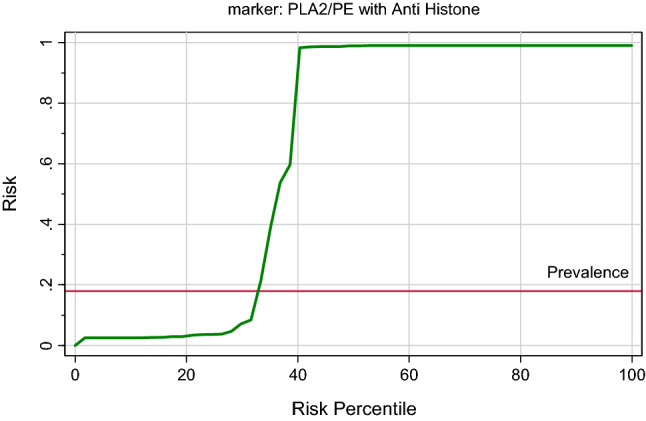
Predictiveness curve of combining of the two parameters for patient group.

Table 1 demonstrates the demographic data of control and ASD participants. Table 2 and Fig. 1 demonstrate the normality of PLA2/PE and anti-histone in plasma of children with ASD compared to gender and age matching healthy controls. It can be easily noticed that while the PLA2/PE ratio was not normally distributed in control, it was normally distributed in ASD patients. Anti-histone was not normally distributed in both groups. Table 3 and Fig. 2 show the min., max., and median of PLA2/PE and anti- histone in plasma of patients with ASD compared to healthy controls. It is clear that while PLA2/PE is significantly higher in ASD patients, anti- histone is significantly lower compared to controls (P = 0.001). Table 4 and Fig. 3 demonstrate the negative correlations between the two measured variables with P of 0.023. Table 5 and Figs. 4 and 5 show the ROC and predectiveness curves of the two measured variables independently while Table 6 and Figs. 6 and 7 demonstrate the combined ROC curves showing the remarkable increase of the AUC and the much improvement of the predictiveness curve of the two combined markers.

**Table 1:** Demographic available data of ASD patients and controls.

Symptom	Autism (40)	Control (40)
Age	5.5 ± 2.2	5.3 ± 2.3
Sex	All males	All males
**GIT**		
Colic	10	1
Constipation	11	1
Diarrhea	2	0
**Allergy**		
Food allergy	13	0
Eye allergy	12	2
Skin allergy	14	3
**Birth complication**		
CS/forceps delivery	19	3
Autoimmunity family history	21	0
**Parental age**		
Average age of father (years)	31.5 (± 6.7)	30.8 (± 5.3)
Average age of mother (years)	35.5 (± 7.2)	34 (± 8.1)

**Table 2:** Normality of data for each group using Shapiro–Wilk Test.

Parameters	Groups	P value
PLA2/PE	Control	0.001
	Patient	0.162
Anti-histone	Control	0.001
	Patient	0.001

The data is normal distributed for P value > 0.05 and not normal distributed for P value <  = 0.05.

**Table 3:** Min., Max. and Median of PLA2/PE and anti- histone in plasma of patients with ASD compared to healthy controls.

Parameters	Groups	N	Min	Max	Median	P value
PLA2/PE	Control	45	0.14	23.74	3.93	0.001
	Patient	40	0.00	246.00	86.47	
Anti- histone	Control	45	0.24	7.70	1.15	0.001
	Patient	40	0.05	8.13	0.37	

The data were statistically represented in terms Minimum, Maximum and Median as a result of normality test (the data of the presented parameters is not normal distributed).

**Table 4:** Spearman’s correlations between PLA2/PE and anti- histone.

Parameters	R (Spearman Correlation)	P value	
PLA2/PE with Anti Histone	-0.300*	0.023	N^a^

*Correlation is significant at the 0.05 level.

^**a**^Negative Correlation.

**Table 5:** ROC-Curve of both variables independently.

Parameters	AUC	Cut-off value	Sensitivity %	Specificity %	P value	95% CI
PLA2/PE	0.900	26.827	90.0%	100.0%	0.001	0.807–0.993
Anti-histone	0.770	0.672	71.1%	75.0%	0.001	0.653–0.887

**Table 6:** Combined ROC results.

Parameters	AUC	Sensitivity%	Specificity%	P value	95% CI
PLA2/PE with anti histone	0.991	94.6%	100.0%	0.001	0.974–1.007

## Discussion

The present study has revealed the usefulness of logistic regression as a simple clinical method with great potential for assisting the diagnosis of ASD. The results of ROC analysis indicated that the combination of anti-histone autoantibodies and PLA2/PE produced remarkably higher AUCs, best sensitivity and specificity for diagnosis of ASD. This could help to understand and interpret the relationship between different recorded markers.

A majority of diseases are triggered by imperfections in signalling pathways. The nature of these imperfections and how they are induced and interacted differs extremely. The notion of signalosome alteration and disease offers a frame for considering how defects in signalling pathways can result in disease. It is useful to separate these defects into phenotypic and genotypic remodelling of the signalosome. In case of phenotypic remodelling, the behaviour of the cells is changed leading to dysfunction and finally to disease. Clearly, there is an urgent need to understand how phenotypic changes in signalosome are interacting to clinically present disease states. Understanding the overlapping etiological mechanisms involved in a complex disorder such as ASD is a crucial challenge in recent biomedical research. Despite the increasing prevalence of ASD, their brain phenotypes remain unclear. Atypical levels of phospholipids, lipid mediators, glutamate/glutamine, creatine /phosphocreatine measured compounds suggest that neuron or glial density, mitochondrial energetic metabolism, and/or inflammation contribute to ASD neuropathology^41^. This might help to design better therapies^41,42^. Signalosomes are protein complexes that undergo assembling to form large supramolecular complexes that increase the local concentration and signalling activity of their individual components. It is well documented that conjugation of PE with selective proteins is involved in membrane closure, and autophagy as a catabolic process that is largely regulated by extracellular and intracellular signalling pathways and contributed to multiple neurological disorders among which is ASD^43–46^.

The evidence that the immune dysfunction expected plays a role in the etiology/pathophysiology of ASD is significant^47^. In the current study while the PLA 2 /PE ratio was dramatically increased level (P = 0.001), anti-histone was significantly reduced (P = 0.001) in the plasma of autistic patients compared to healthy controls (Tables 2 and 3 and Figs. 1 and 2). As the main esterase participating in the synthesis of several inflammatory mediators, cPLA2 has been established to play vast roles in oxidative stress and autoimmune in the neurological disorders^48^. Children with autism are recognized to have elevated phospholipase A2 (PLA 2) activity compared to their matched healthy control^10,49^. It has been suggested that the instability noticed in fatty acid concentrations may be because of an increase in PLA 2 activity concomitant with much lower phospholipids possibly in connection with the high oxidative stress observed in autistic patients^50^.

The mechanisms that bring about the unusual functions of antihistone and how these impacts on neurological pathogenesis stay inadequately understood. In the present study, the increase of PLA2/PE can easily relate to the alteration of membrane phospholipids and multiple lipid mediators in ASD^51–55^. Phospholipids in the brain are rich with polyunsaturated fatty acids (PUFAs), and PLA2s are the key enzymes catalysing the hydrolysis of the PUFAs in the sn-2 position of the glycerol moiety. Omega 6 arachidonic acid (AA) as a substrate for cyclooxygenases and lipoxygenases, serves as the precursor for synthesis of eicosanoids and prostanoids that mediate a wide variety of inflammatory responses^9,10,56^. Conversely, Omega 3 DHA is effective in mediating anti-inflammatory and neuroprotective responses^57^ and is the precursor for biosynthesis of neuroprotectin D (NPD1) and resolvins^58–60^.

In the present study, the logistic regression combination recorded between anti-histone as anti-nuclear antibodies with PLA2/PE demonstrating a much higher AUC value of 0.991, higher sensitivity, and specificity (Table 6 and Fig. 6), compared to the independent ROC-AUCs of each variable (0.77 & 0.900) (Table 5 and Fig. 4) was interesting because it could help to highlight a defected signalosome in ASD which can be targeted as early intervention strategy.

Although research has focused on the identification of genetic abnormalities, emerging studies increasingly suggest that immune dysfunction as a possible risk factor contributing to the neurodevelopmental discrepancies observed in ASD. Edmiston et al.^61^, extensively studied the relationship between autoimmunity and autoantibodies and ASD. They highlighted the involvement of maternal anti-brain autoantibodies, which are supposed to enter the fetal compartment during pregnancy, as a risk factor for developing ASD and are anticipated to contribute to early neurodevelopmental impairments in the developing fetus, later clinically presented as autistic phenotypes^52,62,63^. The remarkable reported lower anti-histone as nuclear autoantibodies in the plasma of patients with autism compared to healthy control (Table 3 and Fig. 2), could be concomitant with much higher level within the brain, a suggestion that can find support through considering the recorded negative spearman’s correlation (Table 4 and Fig. 3). This can be attributed to the fact that maternal antibodies have greater access to the fetal brain due to increased permissiveness of the blood–brain barrier (BBB) during gestation^64^. Rossi et al.^65^ through the use of immunohistochemistry can visualize not only the presence or absence of anti-brain autoantibodies, but also the specific brain profiles to which they drag. They offer a convincing evidence for several brain-reactive antibodies in plasma from individuals with ASD. This might help to support the suggested influx of anti-histone autoantibodies from plasma to brain through the disrupted BBB as autistic phenotypic remodelling^66,67^.

**Figure 8:**
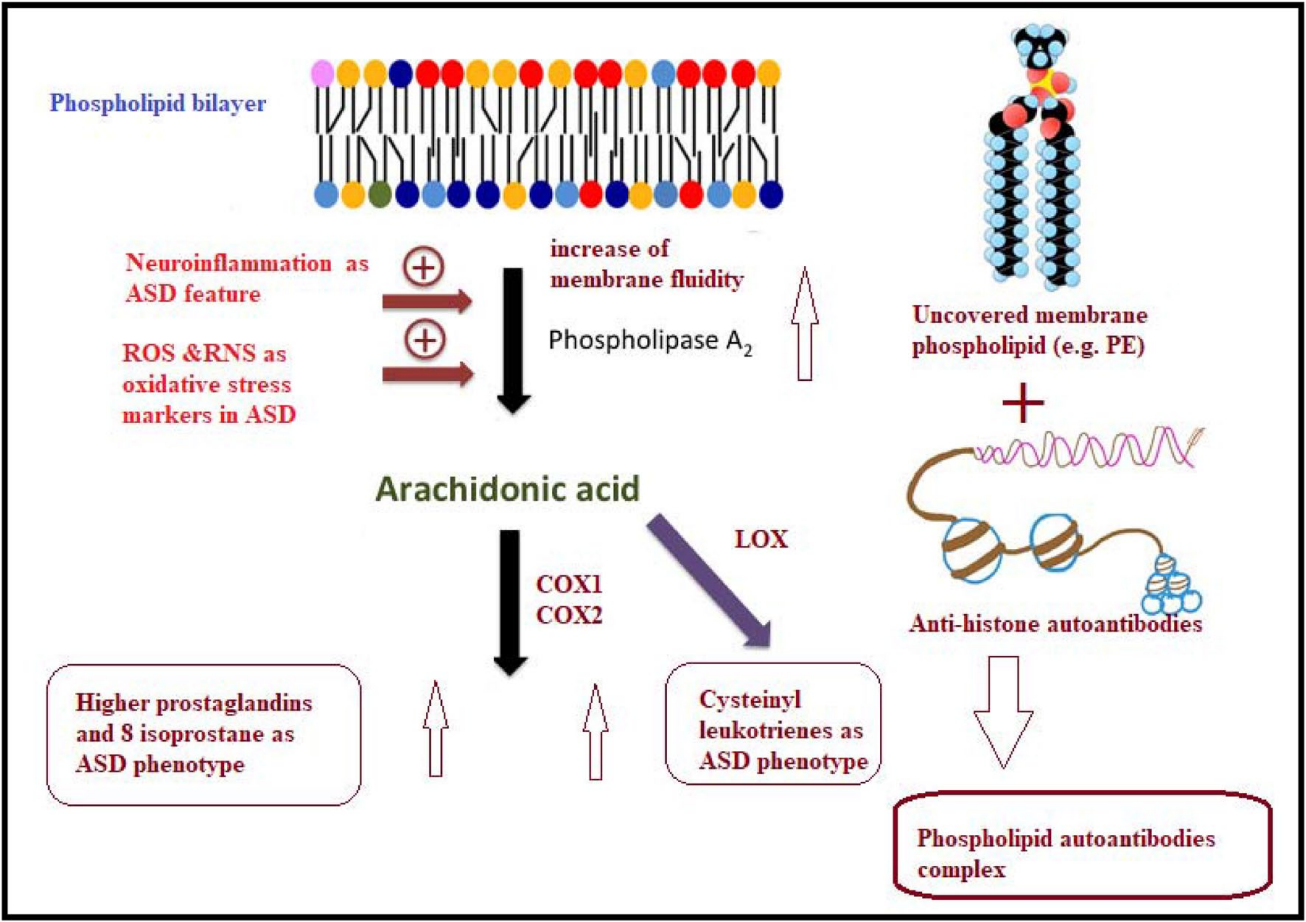
Illustration of the suggested relationship between combined anti-histone autoantibodies, and PLA2/PE relative ratio in inducing neuronal damage. In response to neuroinflammation and oxidative stress, induced elevation of PLA2 as phospholipid hydrolysing enzyme can trigger the elevation of pro-inflammatory lipid mediators (Cysteinyl leukotrienes, and prostaglandins) as ASD phenotype. Suggested influx of anti-histone autoantibodies from plasma to brain through the disrupted BBB induces the formation of PE-anti-histone autoantibodies complex which increases anti-phospholipid antibody levels and greatly affects membrane fluidity.

The importance of combining ROC model between anti-histone and PLA2/PE (Fig. 6) was ascertained with the remarkable improvement of the predictiveness curve of the two combined variables (Fig. 7) relative to the predictiveness curves of each independent variable (Fig. 5). In order to understand the pathological effects of anti-histone in ASD, it is very interesting to know that binding of these autoantibodies to brain structures requires cell destruction, release of antigen and formation of immune complexes. Pereira et al.^31^ suggest that anionic phospholipids among which are PE might be another target structure for these immune complexes. Although these phospholipids are known to be present in the inner part of the cell membrane, they may be uncovered under certain conditions such as activation of neuron and microglial cells as another autistic phenotype^9,68^. This phenomenon of phospholipid-autoantibodies complexing was recently ascertained by the work of Nalli et al.^69^ which demonstrates that anti-phospholipid antibodies analysed by line immunoassay (LIA) can recognize different proteins epitopes depending on to which phospholipids the protein is binding. The obtained higher AUC of combined ROC for anti-histone and PLA2/PE (AUC of 0.991) can find great support in the previous work of Samuelsen et al.^70^ which indicate high prognostic and scientific potential of cross-linked phospholipid-protein conjugates. Moreover, the finding of the present study can find more support in the previous work of Careaga et al.^71^ showing an increased anti-phospholipid antibody levels in young children with ASD that is directly related to the severity of the disorder. In statistics, negative correlation is a relationship between two variables in which one variable decreases as the other increases, and vice versa. A perfect negative correlation means the relationship that occurs between two variables is accurately opposite all of the time. Base on this fact the recorded negative correlation between PLA2/PE and anti-histone autoantibodies with a satisfactory value of − 0.3 could explain and support the remarkable increase of ROC AUCs from 0.900, and 0.77 for independent variables to 0.991 for the two combined variables. Up to the suggested influx of anti-histone autoantibodies from plasma to brain, much higher brain anti-histone autoantibodies, could be contributed in the impairment of membrane fluidity as etiological mechanism of ASD presented by altered PLA2/PE and thus combining both variable could have much higher diagnostic accuracy^72^. Up to the discussed data, the suggested relationship between anti-histone autoantibodies and PLA2/PE relative ratio in inducing neuronal damage is illustrated in Fig. 8.

## Conclusion

This study highlighted the importance of anti-histone autoantibodies as marker of auto-immunity, and inter-related PLA2/ PE ratio as two potential new targets for understanding the mechanisms involved in the pathogenicity of ASD. Although there are reports on autoimmunity family history in ASD, the presence of only 50% (21/40) of the participants with family history of autoimmunity might affect the generalizability of the presented data which could be considered as limitation of this study.

This preliminary findings support the importance of further study of the biological impact of autoantibodies and their association with other etiological mechanisms, behavioural, and cognitive impairments in children with ASD.

## Supplementary Information

Supplementary Information.

## Data Availability

Raw data can be available on request.

## Abbreviations

ASD: Autism spectrum disorder
ADOS: Autism diagnostic observation schedule
AA: Arachidonic acid
AUC: Area under the ROC curve
BBB: Blood brain barrier
COX1: Cyclo-oxygenase 1
COX2: Cyclo-oxygenase 2
DHA: Docosahexaenoic acid
GABA: Gamma-aminobutyric acid
iNOS: Inducible nitric oxide synthase
LOX: Lipo-oxygenase
NOX2: NADPH oxidase
NPD1: Neuroprotectin D
ROC: Receiver operating characteristic
PUFA: Polyunsaturated fatty acids
PLA2: Phospholipase A2
PE: Phosphatidylethanolamine
PC: Phosphatidylcholine
ROS: Reactive oxygen species
SLE: Systemic lupus erythematosus
