# Author Correction: Assessment of a combination of plasma anti-histone autoantibodies and PLA2/PE ratio as potential biomarkers to clinically predict autism spectrum disorders

**DOI:** 10.1038/s41598-022-18827-z

**Published:** 2022-08-25

**Authors:** Afaf El-Ansary, Mona Al-Onazi, Abdulrahman M. Alhowikan, Mashael A. Alghamdi, Laila Al-Ayadhi

**Affiliations:** 1 Central Research Laboratory, Female Center for Medical Studies and Scientific Section, King Saud University, P.O Box 22452, Riyadh, 11495 Saudi Arabia; 2 Department of Biochemistry, College of Science, King Saud University, Riyadh, Saudi Arabia; 3 Department of Physiology, Faculty of Medicine, King Saud University, Riyadh, Saudi Arabia; 4 Department of Chemistry, Imam Mohammad Ibn Saud Islamic University (IMSIU), P.O. Box. 90950, Riyadh, 11623 Saudi Arabia; 5 Autism Research and Treatment Center, Riyadh, Saudi Arabia

Correction to: *Scientific Reports* 10.1038/s41598-022-17533-0, published online 03 August 2022

The original version of this Article contained an error in the spelling of the author Mashael A. Alghamdi which was incorrectly given as Mashael Al-Ghamdi.

The original Article has been corrected.

