# Supplementary material for: Assessment of a combination of plasma anti-histone autoantibodies and PLA2/PE ratio as potential biomarkers to clinically predict autism spectrum disorders
Source: Sci Rep. 2022 Aug 3;12:13359. doi: 10.1038/s41598-022-17533-0 (PMC9349315; doi:10.1038/s41598-022-17533-0)
Supplement: Supplementary file 1 — Supplementary Information. [file 41598_2022_17533_MOESM1_ESM.docx]

**Table S1:** Area under the ROC curves of the independent 24 variables

| Parameters | AUC | Cut-off value | Sensitivity % | Specificity % | P value | 95% CI |
| --- | --- | --- | --- | --- | --- | --- |
| PGE2 pg/ml | 1.000 | 10.916 | 100.0 % | 100.0 % | 0.001 | 1.000 - 1.000 |
| PGE2-EP2 pg/ml | 0.875 | 3697.443 | 77.5 % | 80.0 % | 0.001 | 0.802 - 0.948 |
| PGES ng/ml | 1.000 | 151.281 | 100.0 % | 100.0 % | 0.001 | 1.000 - 1.000 |
| CPLPA2 ng/ml | 1.000 | 1.279 | 100.0 % | 100.0 % | 0.001 | 1.000 - 1.000 |
| 8-Isoprostane pg/ml | 1.000 | 55.049 | 100.0 % | 100.0 % | 0.001 | 1.000 - 1.000 |
| COX-2 ng/ml | 0.776 | 8.784 | 41.5 % | 100.0 % | 0.001 | 0.676 - 0.876 |
| Cysteinyl leukotrienes ng/ml | 0.896 | 0.463 | 86.2 % | 90.0 % | 0.001 | 0.797 - 0.995 |
| PE mmol/L | 0.960 | 0.043 | 95.0 % | 100.0 % | 0.001 | 0.904 - 1.016 |
| PS mmol/L | 0.997 | 0.062 | 97.5 % | 100.0 % | 0.001 | 0.991 - 1.003 |
| PC mmol/L | 0.996 | 1.447 | 100.0 % | 97.4 % | 0.001 | 0.986 - 1.005 |
| PLA2/PE | 0.900 | 26.827 | 90.0 % | 100.0 % | 0.001 | 0.807 - 0.993 |
| PLA2/PS | 0.900 | 20.578 | 90.0 % | 100.0 % | 0.001 | 0.807 - 0.993 |
| PLA2/PC | 0.900 | 0.968 | 90.0 % | 100.0 % | 0.001 | 0.807 - 0.993 |
| COX/PGE2 | 0.808 | 0.252 | 57.1 % | 97.5 % | 0.001 | 0.716 - 0.900 |
| PGE2/CYL | 0.966 | 18.458 | 96.6 % | 100.0 % | 0.001 | 0.899 - 1.032 |
| PLA2/cox | 0.873 | 0.205 | 85.4 % | 85.0 % | 0.001 | 0.785 - 0.960 |
| PGES/PGE2 | 0.938 | 14.394 | 85.7 % | 90.0 % | 0.001 | 0.886 - 0.989 |
| Anti-Histone | 0.770 | 0.672 | 71.1 % | 75.0 % | 0.001 | 0.653 - 0.887 |
| Anti-Nucleosome | 0.830 | 0.340 | 96.9 % | 64.3 % | 0.001 | 0.692 - 0.969 |
| Anti-mitochondrial antibodies (AMA-M2) | 0.587 | 0.112 | 40.7 % | 87.5 % | 0.294 | 0.433 - 0.741 |
| Human Alpha Synuclein | 0.609 | 15.744 | 72.4 % | 55.0 % | 0.124 | 0.473 - 0.745 |
| Human Indian Hedgehog Homolog (IHH) | 0.529 | 0.540 | 91.7 % | 39.1 % | 0.694 | 0.366 - 0.692 |
| Human Neuroligin-4 x linked | 0.655 | 5.337 | 82.6 % | 55.6 % | 0.145 | 0.423 - 0.886 |
| Human nueroligin-3 | 0.885 | 0.216 | 76.5 % | 100.0 % | 0.001 | 0.807 - 0.962 |

**Continued Table S1: Area under the ROC curves of the possible combinations between two variables.**

| Parameters | AUC | Sensitivity % | Specificity % | P value | 95% CI |
| --- | --- | --- | --- | --- | --- |
| PGE2-EP2 pg/ml with COX-2 ng/ml | 0.921 | 97.4 % | 72.5 % | 0.001 | 0.863 - 0.978 |
| PGE2-EP2 pg/ml with PE mmol/L | 0.957 | 87.5 % | 97.4 % | 0.001 | 0.895 - 1.019 |
| PGE2-EP2 pg/ml with COX/PGE2 | 0.940 | 87.5 % | 87.5 % | 0.001 | 0.892 - 0.988 |
| PGE2-EP2 pg/ml with PLA2/cox | 0.951 | 87.2 % | 97.5 % | 0.001 | 0.905 - 0.996 |
| PGE2-EP2 pg/ml with PGES/PGE2 | 0.960 | 100.0 % | 85.0 % | 0.001 | 0.922 - 0.998 |
| PGE2-EP2 pg/ml with Human Alpha Synuclein | 0.917 | 88.5 % | 80.0 % | 0.001 | 0.852 - 0.982 |
| COX-2 ng/ml with PE mmol/L | 0.964 | 90.9 % | 100.0 % | 0.001 | 0.905 - 1.023 |
| COX-2 ng/ml with PLA2/PE | 0.977 | 97.0 % | 100.0 % | 0.001 | 0.934 - 1.021 |
| COX-2 ng/ml with PLA2/PS | 0.977 | 97.0 % | 100.0 % | 0.001 | 0.934 - 1.021 |
| COX-2 ng/ml with PLA2/PC | 0.978 | 97.0 % | 100.0 % | 0.001 | 0.936 - 1.021 |
| COX-2 ng/ml with PLA2/cox | 0.958 | 92.7 % | 100.0 % | 0.001 | 0.902 - 1.014 |
| COX-2 ng/ml with PGES/PGE2 | 0.944 | 92.5 % | 90.0 % | 0.001 | 0.894 - 0.994 |
| COX-2 ng/ml with Anti-Nucleosome | 0.912 | 73.3 % | 100.0 % | 0.001 | 0.830 - 0.994 |
| COX-2 ng/ml with Human Alpha Synuclein | 0.758 | 59.3 % | 87.5 % | 0.001 | 0.635 - 0.882 |
| Cysteinyl leukotrienes ng/ml with COX/PGE2 | 0.950 | 85.7 % | 95.0 % | 0.001 | 0.892 - 1.008 |
| Cysteinyl leukotrienes ng/ml with PGE2/CYL | 0.991 | 96.6 % | 100.0 % | 0.001 | 0.972 - 1.010 |
| Cysteinyl leukotrienes ng/ml with PLA2/cox | 0.945 | 96.4 % | 85.0 % | 0.001 | 0.871 - 1.018 |
| PE mmol/L with COX/PGE2 | 0.967 | 93.9 % | 100.0 % | 0.001 | 0.909 - 1.024 |
| PE mmol/L with PLA2/cox | 0.953 | 97.0 % | 92.3 % | 0.001 | 0.905 - 1.002 |
| PE mmol/L with PGES/PGE2 | 0.970 | 97.0 % | 92.3 % | 0.001 | 0.924 - 1.015 |
| PLA2/PE with Anti Histone | 0.991 | 94.6 % | 100.0 % | 0.001 | 0.974 - 1.007 |
| COX/PGE2 with PGES/PGE2 | 0.946 | 88.1 % | 92.5 % | 0.001 | 0.899 - 0.994 |
| COX/PGE2 with Anti-Nucleosome | 0.898 | 88.1 % | 92.5 % | 0.001 | 0.785 - 1.010 |
| PLA2/cox with PGES/PGE2 | 0.973 | 92.5 % | 92.5 % | 0.001 | 0.943 - 1.002 |
| PLA2/cox with Anti-Nucleosome | 0.900 | 80.0 % | 92.9 % | 0.001 | 0.808 - 0.992 |

**Continued Table S1: Area under the ROC curves of the possible combinations between three variables.**

| Parameters | AUC | Sensitivity % | Specificity % | P value | 95% CI |
| --- | --- | --- | --- | --- | --- |
| PGE2-EP2 pg/ml with COX-2 ng/ml with PLA2/cox | 0.989 | 97.4 % | 97.5 % | 0.001 | 0.971 - 1.007 |
| PGE2-EP2 pg/ml with COX-2 ng/ml with Human Alpha Synuclein | 0.938 | 84.6 % | 92.5 % | 0.001 | 0.882 - 0.993 |
| PGE2-EP2 pg/ml with PE mmol/L with COX/PGE2 | 0.962 | 90.6 % | 100.0 % | 0.001 | 0.903 - 1.020 |
| PGE2-EP2 pg/ml with PE mmol/L with PLA2/cox | 0.982 | 93.8 % | 94.9 % | 0.001 | 0.959 - 1.005 |
| PGE2-EP2 pg/ml with COX/PGE2 with PGES/PGE2 | 0.974 | 92.5 % | 95.0 % | 0.001 | 0.947 - 1.001 |
| PGE2-EP2 pg/ml with PLA2/cox with PGES/PGE2 | 0.983 | 89.7 % | 97.5 % | 0.001 | 0.962 - 1.003 |
| COX-2 ng/ml with PLA2/cox with PGES/PGE2 | 0.987 | 95.0 % | 100.0 % | 0.001 | 0.965 - 1.009 |
| PE mmol/L with COX/PGE2 with PGES/PGE2 | 0.979 | 97.0 % | 92.3 % | 0.001 | 0.952 - 1.006 |
| PE mmol/L with PLA2/cox with PGES/PGE2 | 0.990 | 93.8 % | 97.4 % | 0.001 | 0.975 - 1.006 |
